# The Antimicrobial Peptide 1018-K6 Interacts Distinctly with Eukaryotic and Bacterial Membranes, the Basis of Its Specificity and Bactericidal Activity

**DOI:** 10.3390/ijms232012392

**Published:** 2022-10-16

**Authors:** Rosa Luisa Ambrosio, Catalina Ana Rosselló, Doralicia Casares, Gianna Palmieri, Aniello Anastasio, Pablo V. Escribá

**Affiliations:** 1 Department of Veterinary Medicine and Animal Production, Unit of Food Hygiene, University of Naples Federico II, Via Delpino 1, 80137 Naples, Italy; 2 Laboratory of Molecular Cell Biomedicine, University of the Balearic Islands, 07122 Palma, Spain; 3 Laminar Pharmaceuticals, 07121 Palma, Spain; 4 Institute of Biosciences and BioResources, National Research Council (IBBR-CNR), 80131 Napoli, Italy; 5 Materias S.R.L., Corso N. Protopisani 70, 80146 Naples, Italy

**Keywords:** antimicrobial peptide, *Salmonella* spp., *Staphylococcus* spp., food preservation, membrane lipid therapy (melitherapy)

## Abstract

Since penicillin was discovered, antibiotics have been critical in the fight against infections. However, antibiotic misuse has led to drug resistance, which now constitutes a serious health problem. In this context, antimicrobial peptides (AMPs) constitute a natural group of short proteins, varying in structure and length, that act against certain types of bacterial pathogens. The antimicrobial peptide 1018-K6 (VRLIVKVRIWRR- NH2) has significant bactericidal and antibiofilm activity against *Listeria monocytogenes* isolates, and against different strains and serotypes of *Salmonella*. Here, the mechanism of action of 1018-K6 was explored further to understand the peptide–membrane interactions relevant to its activity, and to define their determinants. We combined studies with model synthetic membranes (liposomes) and model biological membranes, assessing the absorption maximum and the quenching of 1018-K6 fluorescence in aqueous and lipid environments, the self-quenching of carboxyfluorescein, as well as performing lipid sedimentation assays. The data obtained reflect the differential interactions of the 1018-K6 peptide with eukaryotic and prokaryotic membranes, and the specific interactions and mechanisms of action in the three prokaryotic species studied: *Salmonella* Typhimurium^2GN^, *Escherichia coli*^3GN^, and *Staphylococcus aureus*^3GP^. The AMP 1018-K6 is a candidate to prevent (food preservation) or treat (antibiotic use) infections caused by certain pathogenic bacteria, especially some that are resistant to current antibiotics.

## 1. Introduction

Since penicillin was discovered [1], antibiotics have been critical to fight infection. Molecules of diverse structure that display selective toxicity to microorganisms but not humans have saved millions of lives. However, antibiotic misuse has led to the appearance of drug resistance, which currently constitutes a serious health problem [2,3]. In the past few years, the lipid bilayer has been used as a target for therapeutic interventions [4], and since pathogenic bacteria are also bound by a lipid bilayer, this approach could be extended to protection against these microorganisms. Human cell membranes are characterized by a high phosphatidylcholine (PC) and sphingomyelin (SM) content, mainly in the outer monolayer, with relatively high amounts of phosphatidylethanolamine (PE) and phosphatidylserine (PS) in the inner (protoplasmic) monolayer of the plasma membrane [5,6]. By contrast, the plasma membrane of many pathogenic bacteria differs markedly from that of human cells, mainly formed by PE and negatively charged phospholipids (e.g., [PG] or cardiolipin [CL], [7]). Therefore, ligands that selectively bind to the membrane of pathogenic organisms and that have cytocidal effects would be of interest to develop antibacterial compounds. In this context, antimicrobial peptides (AMPs) constitute a natural group of short proteins with distinct structures and lengths, each of which acts against certain types of bacterial pathogen [8,9,10,11,12]. The structures and mechanisms of action of these AMPs differ from those of more conventional antibiotics, which could allow them to elude the resistance strategies used by pathogenic microorganisms. In this context, AMPs have been associated with the immune systems of both prokaryotic and eukaryotic organisms [13,14].

Cationic AMPs (CAMPs) that usually carry a net positive charge (+1 to +9) and several hydrophobic amino acids are especially important [15,16,17]. These features allow cationic AMPs to interact with negatively charged bacterial phospholipids. In eukaryotic cell membranes, these lipids are mainly found at the inner monolayer of the plasma membrane (e.g., PS, phosphatidic acid [PA]; phosphatidylinositol [PI]), which might prevent cationic AMPs from interacting with the plasma membrane of human cells, and that could in part explain the preference of these peptides for prokaryotic membranes. In addition, the higher PC and SM content of the outer monolayer produces a densely packed surface that prevents peptides from binding to the lipid bilayer [4].

The wide range of AMP structures reflect the variation in their length, amino acid composition, net charge, secondary structure, etc., features that drive their mainly lytic action against microbes, even though they may display signaling activity when present below lethal levels [18]. Moreover, as different peptides might interact distinctly with given types of lipid bilayer, the lipid bilayer composition will also define the preference of AMPs for any given type of microorganism. AMP-membrane interactions are mainly driven by electrostatic and hydrophobic forces, and the binding of peptides to membranes changes their structure [19]. The rational design of such peptides aims to avoid interactions with human cell membranes while ensuring important interactions with bacterial membranes in order to compromise their integrity. In the case of an interaction with a bilayer, this could occur at the polar membrane surface, or it could involve deeper hydrophobic membrane regions. For example, the interactions of amyloid peptides (Aβ) with neuronal membranes have been studied in depth [20,21]. These Aβ-membrane interactions greatly depend on the length of the amyloid peptide, its polymerization status (oligomers, fibrils, or plaques), and the membrane lipid composition and biophysical properties derived from this [21,22]. Thus, Aβ peptides can extend over the membrane like a carpet, altering the bilayer’s thickness, or they may form transmembrane pores that affect the composition of the neuron’s cytoplasm. In both these situations, the neuron’s physiology is altered, and its survival may be compromised, provoking well-known neurotoxic effects [23]. Similarly, small changes to the primary structure of AMPs (amino acid sequence) will influence their interactions with bacterial membranes, those peptides that penetrate the hydrophobic core of the membrane usually being more disruptive [24].

In general terms, the primary structure of the peptide and the secondary structure generated from this define the antimicrobial activity of AMPs, the design of which aims to ensure their differential interaction with the pathogen and human cell membrane and inducing toxic effects in the former without compromising the integrity of the latter. In this context, the 1018-K6 peptide (VRLIVKVRIWRR-NH2) has proven to be efficacious against *Salmonella* species and it could be used to combat various types of bacteria [25,26,27,28,29,30]. In the present study, we investigated the differential interactions between the 1018-K6 AMP and model membranes (liposomes) formed using (1) commercially available synthetic or natural lipids, or (2) lipids extracted from eukaryotic or bacterial membranes. These studies indicate that 1018-K6 specifically binds to bacterial but not eukaryotic cell membranes, showing specificity for certain bacterial species. In addition, its antibacterial mechanism of action appears to be associated with its binding and mode of interaction with different lipid bilayers. Therefore, 1018-K6 could be used to develop antibiotics that elude bacterial resistance with a view to treat bacterial infections or to preserve food.

## 2. Results

### 2.1. Interaction of the Antimicrobial Peptide 1018-K6 with Model Membranes

The antimicrobial peptide 1018-K6 (VRLIVKVRIWRR-NH2) has significant bactericidal and antibiofilm activity against *Listeria monocytogenes* isolates and *Staphylococcus aureus* isolates, as well as against different *Salmonella* serotypes bacteria [25,26,27,28,29,30]. Here, the mechanism of action of 1018-K6 was explored further to better understand how it interacts with membranes and the relevance of these interactions to the peptide’s activity.

#### 2.1.1. Peptide Binding to Multilamellar Vesicles (MLVs) and Lipid Sedimentation Assays

**Figure 1:**
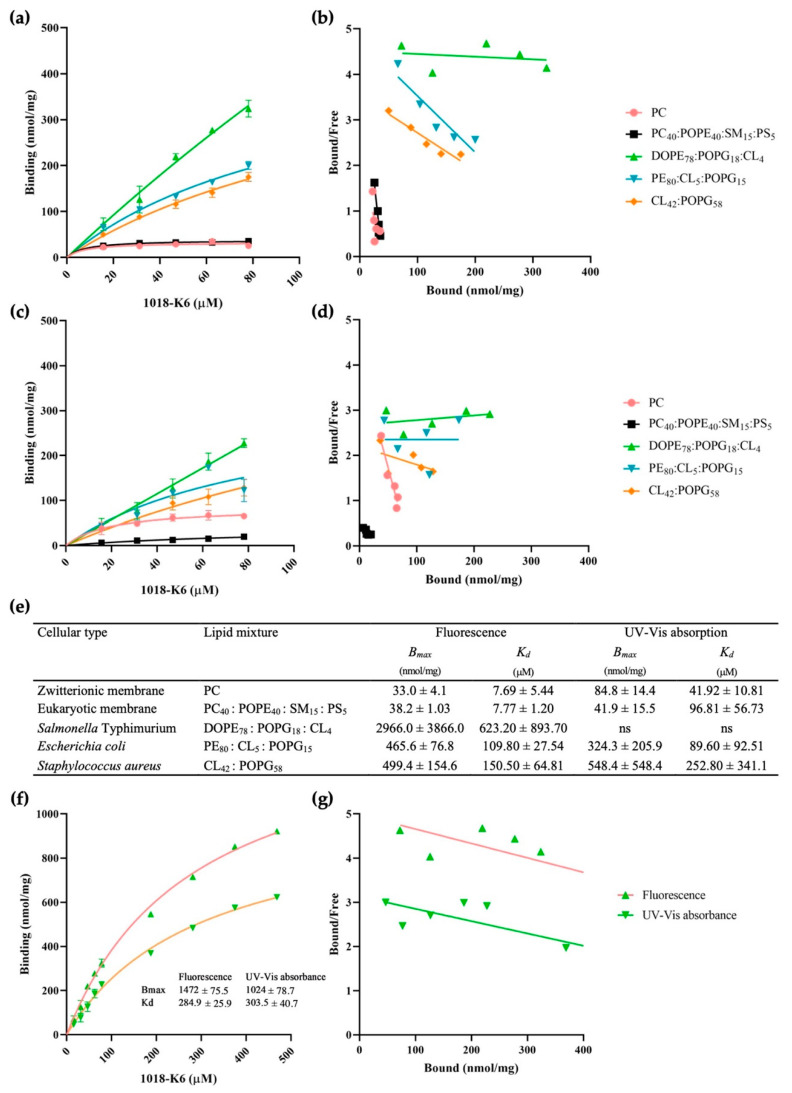
1018-K6 binding to multilamellar vesicles (MLVs) of model membranes (**a**,**c**,**f**) and Scatchard plots of the peptide-MLV interactions (**b**,**d**,**g**). The peptide binding affinity for MLVs was measured by spectroscopic fluorescence (**a**,**b**) and UV-Vis absorption (**c**,**d**), and by calculating the *B_max_* and *K_d_* values (**e**): ns, unsaturated binding curve). Binding curves (including the *B_max_* and *K_d_* values) and Scatchard analyses of the peptide-DOPE_78_:POPG_18_:CL_4_ interaction with increasing peptide concentrations (**f**,**g**). All the measurements are the average of three experiments.

To define the mode of action of this membrane-bound compound, its interaction with model synthetic and biological membranes was assessed by determining the ligand bound to the lipid bilayer or free in the aqueous medium. Model membranes (liposomes) were prepared as described in the Materials and Methods (Section 4.2 and Section 4.3) and the binding parameters of the peptide to pre-formed membranes was evaluated by separating the bound and free peptides by ultracentrifugation [31]. This separation allows the fraction of the peptide partitioned to be calculated, ranging from 0 to 100%. To separate bound peptide from that not bound to lipid bilayers, the samples were centrifuged at high speed and pre-formed MLVs (with no peptide inside) were used to study binding as opposed to the small unilamellar vesicles (SUVs) commonly used in partitioning studies [32]. A Scatchard analysis [33] was used to describe the interactions between peptide and lipids, which allows the dissociation constant *K_d_* (binding affinity) and the number of peptide binding sites, B_max_, to be determined once the bound and the free ligand concentrations were determined experimentally [34,35]. Since lipid membranes are complex systems and the peptide-lipid interaction is controlled by the lipidic ensemble [36], the Scatchard method should be appropriate to analyze this type of interaction if the lipid bilayer is considered as a receptor and the peptide as a ligand [37]. The ability of 1018-K6 to bind to phospholipid bilayers was examined in saturation binding experiments, fitting the experimental data with Equations (1) and (2) described in Section 4.4. The 1018-K6 peptide contains one Tryptophan (Trp) residue that facilitates its characterization using spectroscopic techniques [25]. The concentration of peptides in the aqueous (supernatant-free ligand) and lipidic (pellet-bound ligand) phases was measured using fluorescence emission (Figure 1a,b) and UV-Vis(ual) absorption (Figure 1c,d), and based on the analysis of the Trp signals in both phases.

The behavior of the cationic peptide differed from one type of model membrane to another (Figure 1e), probably influenced by the distinct lipid compositions and principally dependent on the membrane’s surface charge. The first step to understand an AMP’s mechanism of action is to investigate the peptide-lipid binding interactions. Indeed, electrostatic forces attract cationic residues of CAMPs to negatively charged lipids, such as PS, PG, or CL in the target bacterial membrane [38,39,40,41]. 1018-K6 has a net positive charge [25] and the potential interaction of this molecule with lipids containing net negative charges has already been hypothesized. This hypothesis was supported by earlier work [42] in which membranes composed of zwitterionic PC, simulating the bulk fluid phases in the eukaryotic cell, were compared with those that mimic anionic bacterial cells. The receptor density assessment supported a very low affinity of 1018-K6 for membranes with a net neutral charge or with a low negative charge (PC and PC_40_:palmitoyl-oleoyl-phosphatidylethanolamine[POPE]_40_:SM_15_:PS_5_, respectively). Moreover, the 1018-K6 peptide was preferentially bound to model membranes resembling that of *Salmonella* Typhimurium, reaching its highest B_max_ after binding was determined by both spectroscopy methods (Figure 1f,g). Moreover, it was necessary to increase the maximum peptide dose more than 5-fold to carry out saturation binding experiments with this type of membrane (Figure 1f,g). This finding suggests the propensity for the peptide to bind to Gram-negative bacterial cells much more easily than Gram-positive ones. In terms of interactions, it can be argued that the *Salmonella*-like membrane has more binding sites and/or that its affinity is higher than that of the eukaryotic-like membrane. Nevertheless, the values of binding potential (BP = B_max_/*K_d_*) (Table S1) as well as those of *K_d_* (Figure 1e) relative to anionic model membranes were not found to be in line with B_max_ values, which were very encouraging. To this end, it is worth considering the low concentration of peptide used in this study (15.63 to 78.13 µM) and assume that further study by increasing the peptide doses may be needed to achieve the saturation, where all the binding sites (sites for ligand) of anionic model membranes are occupied by the peptide. Indeed, as we can see in Figure 1, all of the binding sites of the zwitterionic model membrane (PC and eukaryotic) were still fully occupied at ~30 µM. In any case, according to the data obtained in absorbance (Figure 1e; Table S1), it is possible to affirm that the affinity of the peptide towards the bacterial model membranes is higher than that obtained towards the eukaryotic model membrane. Moreover, the BP value referring to the experiments with model membranes of *Salmonella* Typhimurium clearly indicate a higher binding of the 1018-K6 peptide toward these liposomes, confirming the theory that the antimicrobial compound seems to prefer the *Salmonella*-like membrane.

The partitioning of the peptide into lipid vesicles was obviously dependent on the MLV composition since the presence of negatively charged lipids (Table 1), such as CL and Palmitoyl-oleoyl-phosphatidylglycerol (POPG), and the nonlamellar-prone lipid dioleoyl-phosphatidylethanolamine (DOPE)/PE, was associated with high *K_p_* values. Again, there was a significant difference (*p* < 0.01) between the coefficient values of the model *E. coli* membrane when measured with different spectroscopic methods. Interestingly, and unlike previously described datasets that focused on B_max_ and *K_d_* values, the affinity of the 12-mer peptide appeared to be stronger for *E. coli* MLVs than the other model bacterial membranes. This observation could be explained by the difference between Equations (1) and (2). The first method calculates B_max_ and *K_d_* values by fitting the data regarding the concentration of peptides detected in the lipidic environment (pellet), while the second evaluates the *K_p_* values by fitting the amount of 1018-K6 present in the aqueous phases (supernatants). Therefore, the absence or a low concentration of lipid in the supernatants may permit the measurement of Trp residues without interference even in *E. coli* MLV samples.

Because many models of AMP mechanisms of action lead to membrane rupture [16], the possible fragmentation of the membrane into peptide-lipid micelles was evaluated. The same samples used to calculate the parameters of peptide affinity to the model membranes were subjected to the modified Fiske protocol to assess the lipid phosphorus concentration (see Section 4.8). Only a very small percentage of the total lipid concentration could be detected in the supernatant of the control samples (0 μM 1018-K6: Figure S1), demonstrating the effectiveness of the separation technique. Moreover, it is worth noting that no significant differences were evident between the MLV samples incubated with increasing peptide doses for 30 min and the controls. Hence, we propose two different hypotheses: (1) the mechanism of action of 1018-K6 involves pore formation according to the “toroidal wormhole” model [16], without causing membrane disruption; (2) an incubation longer than 30 min is necessary to detect the final effect on the membranes.

#### 2.1.2. Peptide Binding to Multilamellar Vesicles (MLVs) from Biological Membranes

The peptide’s interaction with membrane surfaces involves several events, the first of which is the attraction of cationic AMP residues to negatively charged lipids in the bacterial target membrane through electrostatic forces [41]. The resulting approximation of the peptide to the membrane drives changes in its conformation, commonly through a coil-helix transition [43,44], although this is not the case for β-sheet AMPs that have more rigid structures in solution. Following this electrostatic interaction, it is necessary to reach a critical concentration of peptides to induce self-association and full or partial lipid bilayer penetration. Once situated in the membrane core, AMPs can exert their action through different mechanisms [45,46].

**Figure 2:**
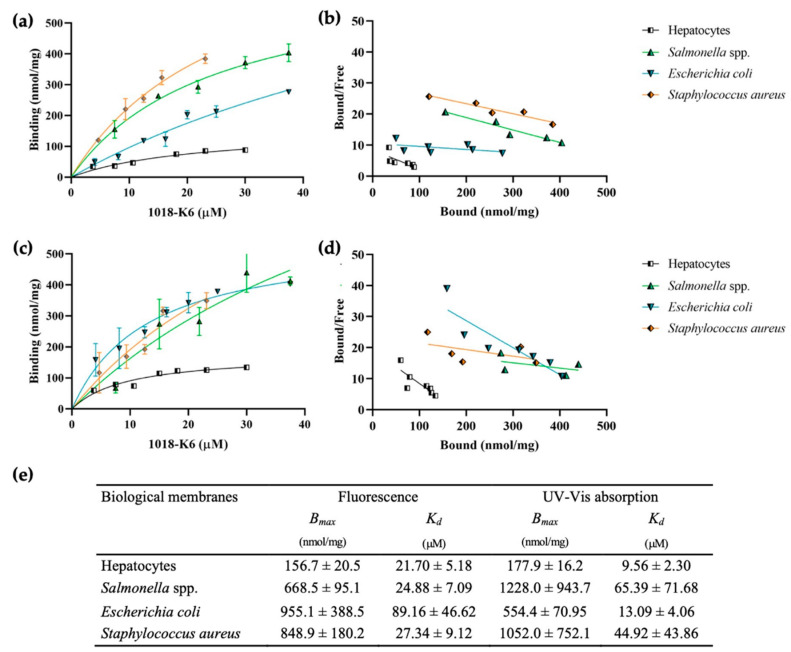
1018-K6 binding to multilamellar vesicles (MLVs) formed with lipids extracted from biological membranes (**a**,**c**) and Scatchard plots of the peptide-MLV interactions (**b**,**d**). The peptide binding affinity for the MLVs was measured using fluorescence (**a**,**b**) and UV-Vis absorption (**c**,**d**), and by calculating the *B_max_* and *K_d_* values (**e**). All the measurements are the average of three experiments.

We can contemplate the binding of 1018-K6 to the membranes of Gram-positive and Gram-negative bacteria as a function of the peptide concentration (Figure 2). In order to simulate these conditions in the MLV studies, the doses of peptide added were calculated as the ratio of the lipid and peptide concentrations. In these membrane binding saturation experiments, biological membranes were prepared from bacterial strains and eukaryotic cells (hepatic rat tissue), with only plasma membranes free of mitochondrial membranes and nuclei used to construct the MLVs. By maintaining the same lipid-peptide ratio used with mimetic membranes (C_L_/C_P_, the ratio between the lipid phosphorus concentration–C_L_– and the peptide concentration–C_P_), membrane saturation was achieved for hepatocytes, *Salmonella* spp. and *S. aureus*, enabling the maximum receptor density and the dissociation constant to be calculated. By contrast, the peptide doses used did not saturate the binding sites of *E. coli* membranes and, therefore, higher concentrations of AMPs were used to correctly calculate the B_max_. As already seen for mimetic membranes, 1018-K6 seems to have greater binding capacity for bacterial compared to eukaryotic cell membranes (Figure 2e). Specifically, the fluorescence measurements underlined higher binding of the antimicrobial peptide to the *E. coli* membranes (Figure 2a), although this observation was not confirmed by the UV-Vis absorption data (Figure 2c). In the light of the earlier results and the behavior with *E. coli* model membranes, the results reinforce the initial hypothesis that the interaction of 1018-K6 might be stronger for bacterial membranes with some preference for *E. coli* lipid bilayers, and the disruption of *E. coli* membranes through the quenching of Trp could be detected. Furthermore, the lipid composition of the two Gram-negative bacterial membranes seems to be more similar than that of the model membranes, such that a difference between the two spectroscopic methods was also noted for *Salmonella* spp. (Figure 2). As far as the *K_d_* and binding potential is concerned (Figure 2e, Table S2), the obtained results pointed out two important aspects: (i) 1018-K6 peptide showed a higher binding toward membranes of Gram-positive and Gram-negative bacteria; (ii) the peptide has higher binding towards biological membranes than those of the model membranes. 

These results support the antimicrobial effect of 1018-K6, adding to the bactericidal and antibiofilm efficacy of the peptide toward strains of *Salmonella* spp. [29] and *Staphylococcus aureus* [28]. In particular, several serotyped wild strains (of *Salmonella* spp.) were selected for study based on their resistance to common antibiotics and the antimicrobial efficacy of 1018-K6 against them was evaluated. Accordingly, this AMP was seen to be able to kill resistant Gram-positive and Gram-negative microorganisms (5 × 10^5^ cfu/mL) at concentrations ≤ 20 μM [28,29]. Moreover, the possible fragmentation of the biological membranes into peptide-lipid micelles was evaluated in these studies (Figure S2).

### 2.2. Fluorescence Properties of 1018-K6 in Aqueous and Lipid Solutions

#### 2.2.1. Blue Shifts in Emission Spectra

**Figure 3:**
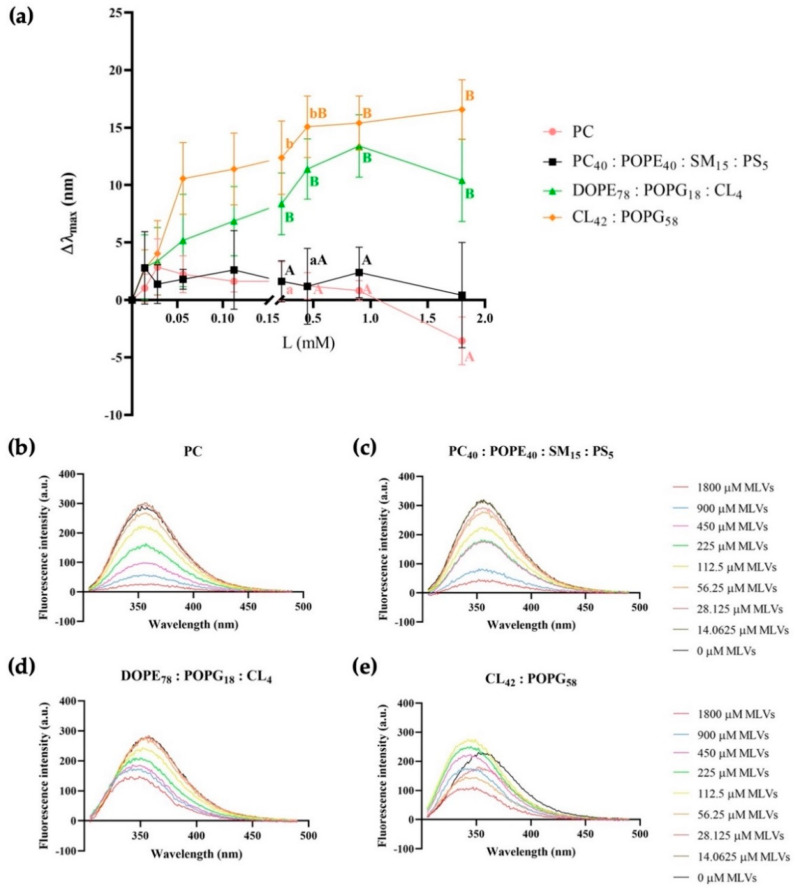
Blue shift in Trp fluorescence of the 1018-K6 AMP in the presence or absence of MLVs (L, concentration up to 2 mM) (**a**) Δλ_max_ shift of AMP in model membranes of zwitterionic lipids (PC, pink), eukaryotic cells (PC_40_:POPE_40_:SM_15_:PS_5_, black), *S.* Typhimurium (DOPE_78_:POPG_18_:CL_4_, green) and *S. aureus* (CL_42_:POPG_58_, orange). The data were analyzed with SPSS version 26 (IBM Analytics, Armonk, NY, USA) and the results were assessed with generalized linear mixed models (GLMMs), comparing the means with the Tukey test. All data are presented as the mean (M) ± standard error (SE): ^A^, ^B^ values differ at *p* < 0.01; ^a^, ^b^ values differ at *p* < 0.05. (**b**–**e**) Fluorescence emission spectra of 1018-K6 before and after the addition of MLVs simulating (**b**) zwitterionic membranes, (**c**) eukaryotic cells, (**d**) *S.* Typhimurium, and (**e**) *S. aureus*.

The peptide 1018-K6 owes its fluorescence to the presence of a Trp residue in its sequence, the aromatic ring of which is sensitive to the environment. Hence, the intrinsic steady-state fluorescence can provide information on the organization and position of peptides in the aqueous environment. As such, an aqueous suspension of 1018-K6 (20 μM) was titrated with increasing concentrations of MLVs (0 to 2 mM of lipid phosphorus) and peptide partition into the membrane was recorded. The 12-mer peptide emission spectra has a characteristic blue shift when titrated with model bacterial membranes (Figure 3) and this shift in the maximum wavelength of emission (Δλ_max_) is associated with the transition of Trp residue from an aqueous to a more hydrophobic environment [36]. The Δλ_max_ shift was greatest when the peptide interacted with the model membrane of *S. aureus* at a lipid phosphorus concentration of 1.8 mM, associated with the specific maximum shift of ~16.55 (Figure 3a). No significant differences were found between the shift of the emission maximum between model membranes from Gram-positive and Gram-negative bacteria, although significant differences among zwitterionic (PC or eukaryotic) and anionic (prokaryotic) membranes were detected from a MLV concentration of 0.225 mM. Finally, the observed reduction in the fluorescence maximum emission at increasing lipid concentrations could be originated by the light scattering caused by the lipid vesicles in the buffer.

#### 2.2.2. Fluorescence Quenching of 1018-K6

Fluorescence quenching has been used widely to gain information regarding molecular interactions and, thus, fluorescence-quenching experiments were performed here using acrylamide and nitromethane quenchers to detect the relative position of the 1018-K6 peptide in the lipid bilayer. Indeed, given the incapacity of the soluble acrylamide quencher to penetrate the lipid bilayer, the Trp residue cannot be quenched when the peptide is in the hydrophobic membrane core. Fluorophore quenching is therefore directly proportional to the concentration of the quencher added and the exposure of Trp residue to the aqueous environment. Thus, only the 1018-K6 peptides not inserted into the membrane will be quenched by acrylamide, which includes free peptide molecules and peptide molecules adsorbed to the surface of the model membrane. By contrast, Trp residues from the 1018-K6 molecules inserted into the lipid bilayer will not be accessible to acrylamide and their fluorescence emission will persist. To evaluate the relative position of the peptide and its interaction with membranes, the quenching of fluorescence emission after titration with acrylamide was performed and *Stern–Volmer* constants (*K_SV_*) were calculated [47].

**Figure 4:**
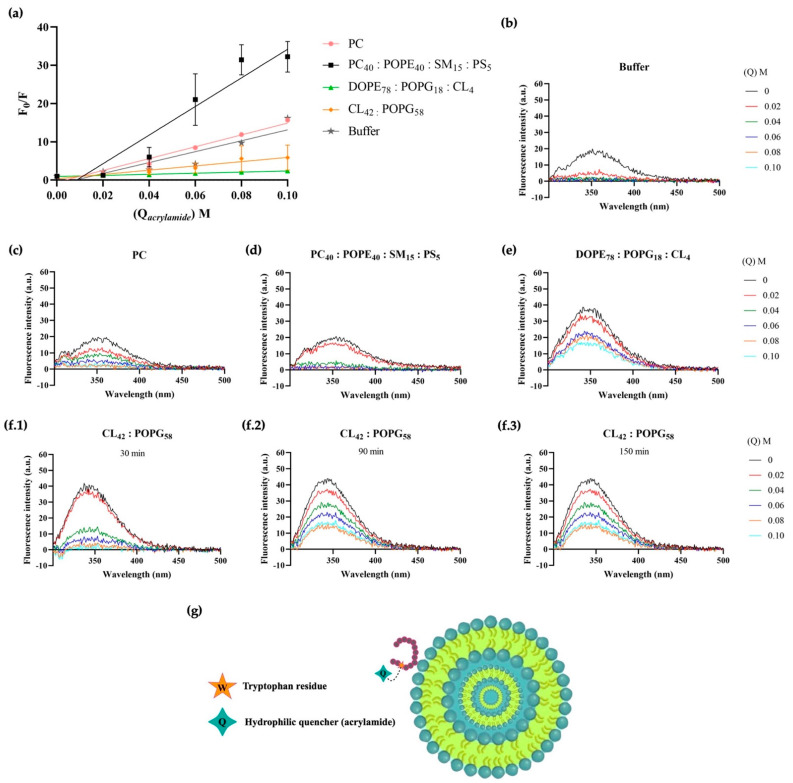
1018-K6 Trp fluorescence quenching by acrylamide in model membranes. (**a**) Stern-Volmer plots for acrylamide quenching of Trp fluorescence when 1018-K6 was maintained in aqueous buffer (1 μM, grey line) or lipidic environments (30 μM): model membranes of zwitterionic lipids (PC, pink line), eukaryotic cells (PC_40_:POPE_40_:SM_15_:PS_5_, black line), *S.* Typhimurium (DOPE_78_:POPG_18_:CL_4_, green line), or *S. aureus* (CL_42_:POPG_58_, orange line). The results are expressed as the mean and standard error of three independent scans 30, 90 and 150 min after peptide:liposomes incubation. (**b**–**f**) Fluorescence spectra of the 1018-K6 AMP in (**b**) buffer, (**c**) zwitterionic lipid, (**d**) eukaryotic cells, (**e**) *S.* Typhimurium and (**f**) *S. aureus* before and after the addition of acrylamide (up to 0.1 M). Details of the peptide spectra in liposomes of CL_42_:POPG_58_, recording the extent of tryptophan quenching depending on the incubation time (**f.1**–**f.3**). (**g**) Non-scale schematic drawing.

Changes in fluorescence intensity of the peptide-MLV solution were recorded as the concentration of acrylamide increased (see Figure 4 and Table 2), although the Trp fluorescence decreased rapidly when the peptide was surrounded by buffer or incubated with zwitterionic model membranes. Indeed, the fluorescence of the free peptide in the buffer was almost completely quenched by adding only 0.02 M of acrylamide, while twice this concentration was needed to quench the Trp emission when the peptides were incubated with model eukaryotic cell membranes. Hence, when the peptide is located at the lipid vesicle–water interface or in the absence of interactions of the AMP molecules with lipids, all the Trp residues appeared to be exposed to quenching. Similarly, the Trp residue of the peptide is more accessible to quenching in the presence of PC membranes relative to other model membranes, indicating there are no deep membrane interactions between 1018-K6 and vesicles resembling eukaryotic membranes.

The quenching efficacy was very low in the presence of DOPE_78_:POPG_18_:CL_4_ membranes, probably due to the deeper insertion of the peptide into the phospholipid bilayer as soon as 30 min after exposure to the membranes (Table 2). Different dynamics were described for *S. aureus* model membranes, into which peptide insertion appeared to be slower than in Gram-negative bacteria. Indeed, 20 min more were previously seen to be required for peptide binding to *S. aureus* model membranes than to *E. coli* model membranes [48]. The importance of the membrane surface potential was also highlighted, such that the surface potential could interfere with the permeation of the peptide into membranes with different lipid compositions.

**Figure 5:**
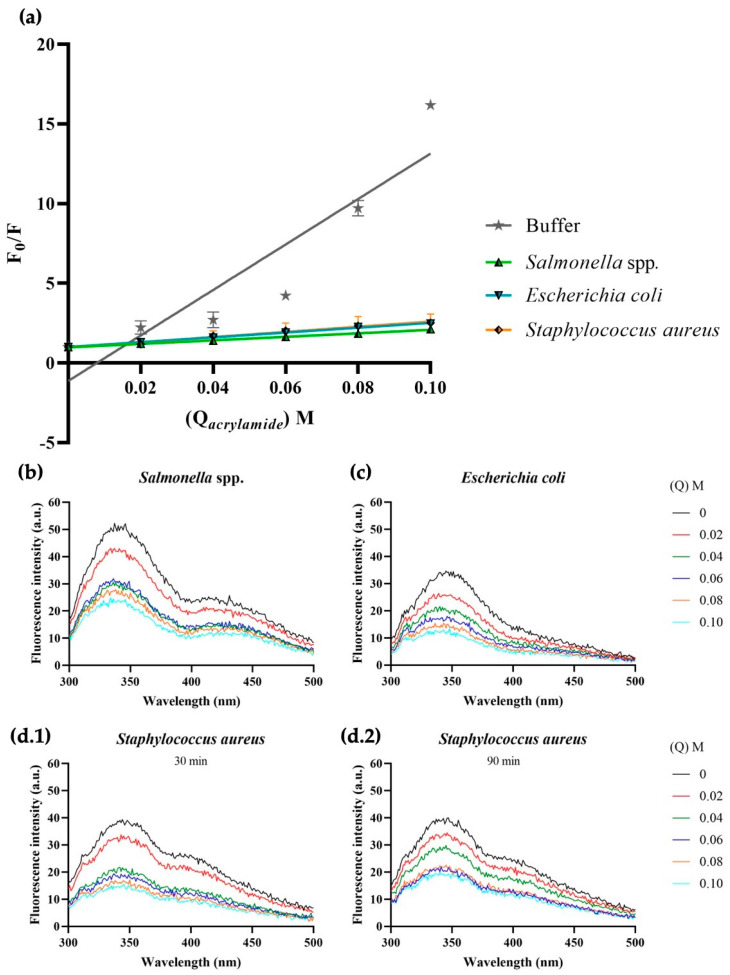
Trp fluorescence quenching of 1018-K6 in biological membranes by acrylamide. (**a**) Stern–Volmer plots of Trp fluorescence acrylamide quenching for 1018-K6 in aqueous buffer (1 μM, grey line) or lipidic environments (30 μM): *Salmonella* spp., *E. coli* or *S. aureus* biological membranes. The results are expressed as the mean and standard error of three independent scans after a 30, 90 or 150 min peptide:liposome incubation. (**b**–**d**) Fluorescence spectra of AMP 1018-K6 partitioned into liposomes of (**b**) *Salmonella* spp., (**c**) *E. coli*, or (**d**) *S. aureus* before and after the addition of acrylamide (up to 0.1 M). Details of the peptide spectra in liposomes of *S. aureus*, recording the extent of tryptophan quenching at different incubation times (**d.1**,**d.2**).

The same quenching behavior induced by acrylamide in model membrane MLVs was replicated in biological membrane MLVs, particularly for *Salmonella* spp. and *S. aureus* (Figure 5). The *K_SV_* values confirmed the higher membrane-binding affinity of 1018-K6 with anionic membrane and they better described the situation with *S. aureus* MLVs (Table 2 and Table 3). Indeed, after 150 min, the constant fell more than 100-fold relative to the first interval, reaching values similar to those for *S.* Typhimurium.

**Figure 6:**
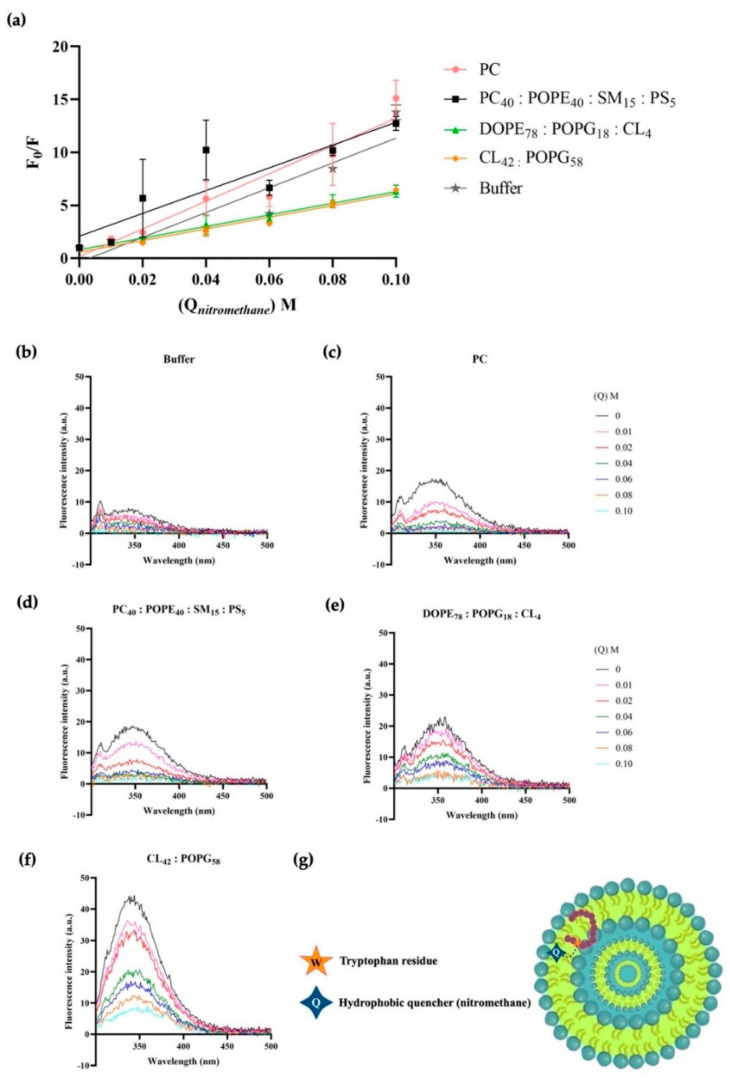
1018-K6 Trp fluorescence quenching by nitromethane in model membranes. (**a**) Stern–Volmer plots for nitromethane quenching of Trp fluorescence from 1018-K6 in aqueous buffer (1 μM, grey line) and lipidic environments (30 μM): model membranes of zwitterionic lipids (PC, pink line), eukaryotic cell (PC_40_:POPE_40_:SM_15_:PS_5_, black line), *S.* Typhimurium (DOPE_78_:POPG_18_:CL_4_, green line) or *S. aureus* (CL_42_:POPG_58_, orange line). The results are expressed as the mean and standard error of three independent scans after a 30, 90 or 150 min peptide:liposome incubation. (**b**–**f**) Fluorescence spectra of the 1018-K6 AMP in (**b**) buffer or (**c**) zwitterionic lipid, (**d**) eukaryotic cell, (**e**) *S.* Typhimurium and (**f**) *S. aureus* membranes before and after the addition of nitromethane (up to 0.1 M). (**g**) Non-scale schematic drawing.

**Figure 7:**
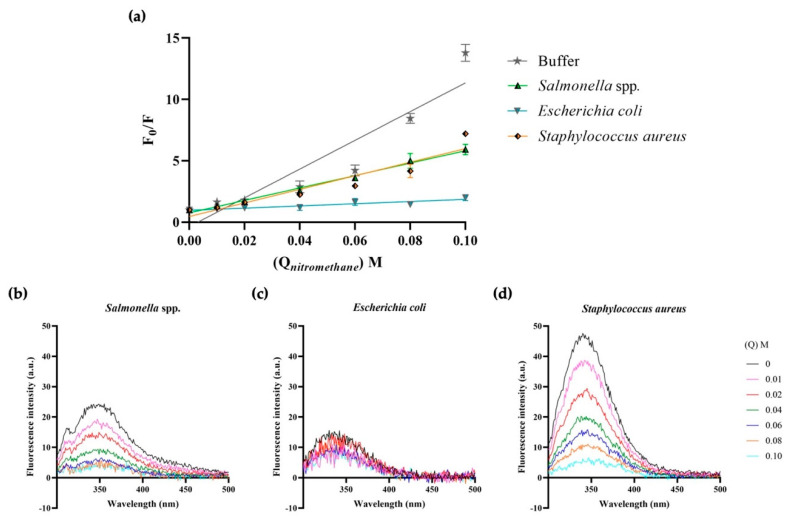
1018-K6 Trp fluorescence quenching in biological membranes by nitromethane. (**a**) Stern–Volmer plots of Trp fluorescence quenching by nitromethane when 1018-K6 is maintained in aqueous buffer (1 μM, grey line) or lipidic environments (30 μM): biological membranes of *Salmonella* spp., *E. coli* or *S. aureus*. The results are expressed as the mean and standard error of three independent scans after a 30, 90 or 150 min peptide:liposome incubation. (**b**–**d**) Fluorescence spectra of AMP 1018-K6 partitioned into liposomes of (**b**) *Salmonella* spp., (**c**) *E. coli*, or (**d**) *S. aureus* before and after the addition of nitromethane (up to 0.1 M).

Nitromethane has been used extensively to quench the fluorescence of polycyclic aromatic hydrocarbons [49] and, thus, it was assessed whether 1018-K6 molecules inserted into membranes were accessible to quenching by nitromethane. Here again, fluorophore quenching is directly related to the concentration of the quencher added and the exposure of the Trp residue to it. Changes in intensity of peptide-MLV fluorescence were recorded by increasing the concentration of nitromethane (see Figure 6 and Table 4). Indeed, the fluorescence results were very similar to those of acrylamide when the peptide was surrounded by buffer or incubated with zwitterionic model membranes. In this situation, fluorescence was quenched almost completely by adding only 0.01 M of nitromethane and twice the dose was needed to quench Trp emission of peptides incubated with eukaryotic cell membrane models. However, complementary to the results obtained with acrylamide using MLVs of model *S.* Typhimurium bacteria, where 0.06 M was needed to reduce the emission by half (Figure 4e), here, only 0.04 M nitromethane was required to reduce the emission by two thirds (Figure 6e). Similar results were obtained with both acrylamide (Figure 5b) and nitromethane (Figure 7b) when MLVs constructed from *Salmonella* spp. biological membranes were studied. This reinforces the idea that the association of 1018-K6 with *S.* Typhimurium is not merely superficial, but rather, that there is partial internalization of the peptide into the bacterial membrane.

The results obtained with nitromethane using MLVs constructed from model and biological *S. aureus* membranes were also consistent with those acquired with acrylamide. Essentially, 0.04 M nitromethane reduced Trp emission by 50% (Figure 6f and Figure 7d, respectively) while 0.04 M acrylamide reduced its emission by 75% in the first 30 min of incubation, even if this effect was diluted after 90 and 150 min (Figure 4f and Figure 5d, respectively). These data confirm the peculiar two-phase interaction of the 1018-K6 peptide with *S. aureus*, initially at a superficial level but later deeper inside the membrane. The *K_SV_* values captured further support this conclusion (Table 4 and Table 5).

### 2.3. The Effect of 1018-K6 on the Permeabilization of Model and Biological Membranes

A fluorescence dequenching assay with 5-carboxyfluorescein (CF) loaded Large Unilamellar Vesicles (CF LUVs) was used as a model system to evaluate the potential pore-forming activity of 1018-K6 [50]. Although the encapsulated CF exhibits fluorescence self-quenching in these LUVs, we hypothesized that 1018-K6 might permeabilize the LUVs derived from certain membrane types, driving the release of CF from these structures and its subsequent dequenching, thereby augmenting the intensity of fluorescence emission in the model. Thus, CF fluorescence dequenching was used as a measure of permeabilization as described in Equation (4). This increase in fluorescence will only occur when the interaction of 1018-K6 with lipid bilayers results in pore formation (≥ 1 nm in diameter) and the vesicle’s contents are released. 

**Figure 8:**
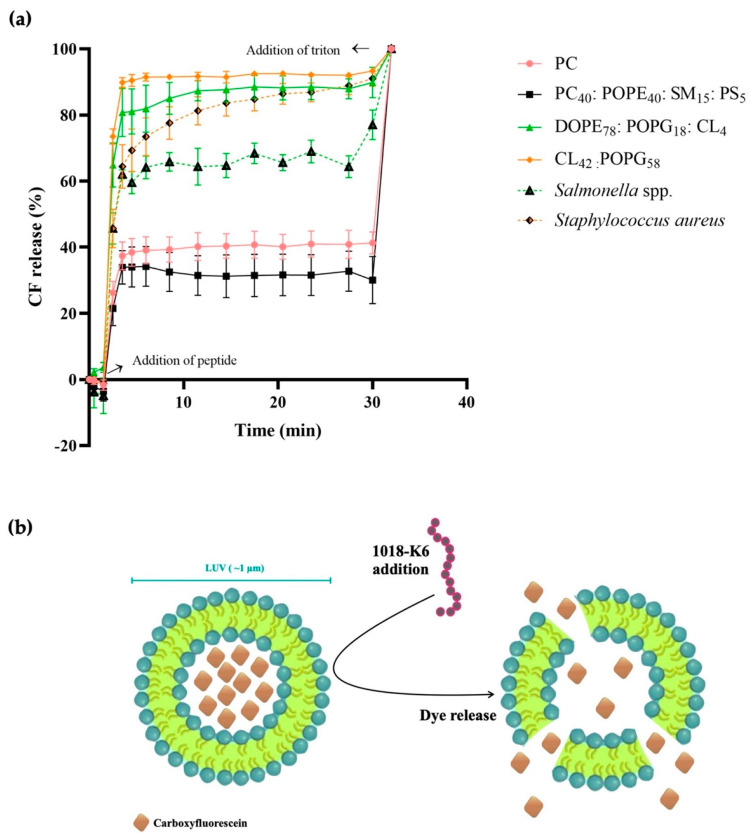
Permeabilization efficiency of AMP 1018-K6 (15 µM). (**a**) The kinetic release of carboxyfluorescein (CF) from LUVs derived from model zwitterionic (PC, pink line), eukaryotic cell (PC_40_:POPE_40_:SM_15_:PS_5_, black line), *S.* Typhimurium (DOPE_78_:POPG_18_:CL_4_, green line) and *S. aureus* (CL_42_:POPG_58_, orange line) membranes, and from biological membranes (*Salmonella* spp. and *S. aureus*). All the data correspond to the mean ± standard error (SE) of three independent experiments. (**b**) Non-scale schematic drawing.

CF fluorescence dequenching occurred rapidly from model bacterial LUVs (CL_42_:POPG_58_ and DOPE_78_:POPG_18_:CL_4_) and their respective biological LUVs (*Salmonella* spp. and *S. aureus*: Figure 8) upon addition of 1018-K6. A peptide dose of 15 µM was sufficient to achieve 90% permeabilization of CL_42_:POPG_58_ LUVs (*S. aureus*) and 80% permeabilization of the DOPE_78_:POPG_18_:CL_4_ LUVs (*S.* Typhimurium), while 80% permeabilization of the *S. aureus* biological LUVs was achieved after 10 min and 65% of the *Salmonella* spp. LUVs. By contrast, zwitterionic membrane (PC) and eukaryotic cell (PC_40_:POPE_40_:SM_15_:PS_5_) LUVs do not exceed 40% permeabilization at this peptide dose.

### 2.4. Effect of 1018-K6 on Vesicle Aggregation

**Figure 9:**
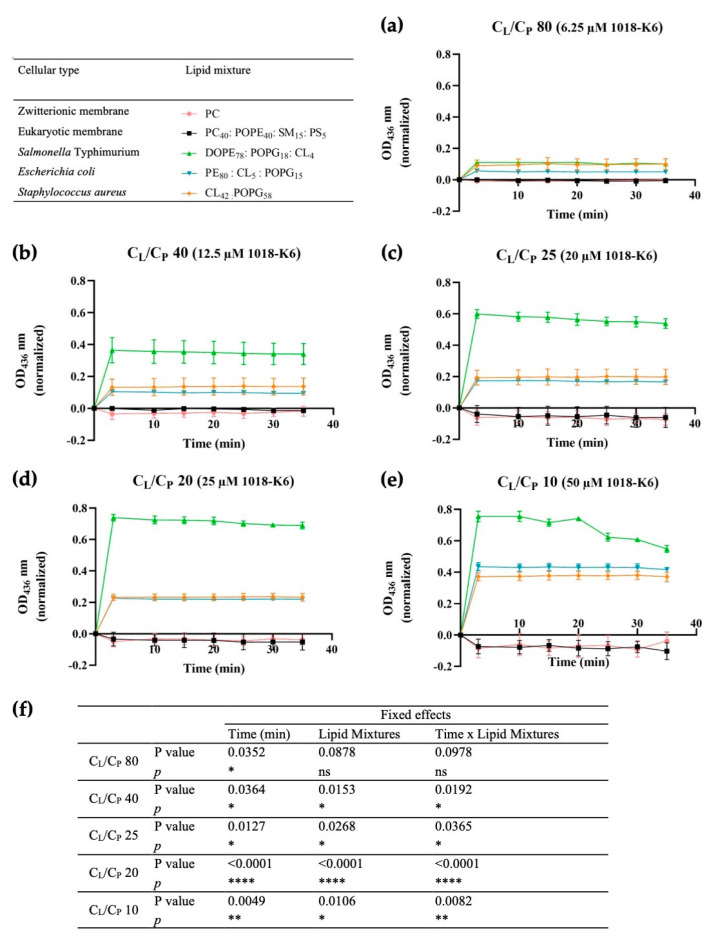
Kinetics of MLV aggregation in the presence of 1018-K6. (**a**) 6.25 µM; (**b**) 12.5 µM; (**c**) 20 µM; (**d**) 25 µM; (**e**) 50 µM. Model zwitterionic (pink), eukaryotic cell (black), *S.* Typhimurium (green), *E. coli* (blue) or *S. aureus* (orange) membranes were used at a concentration of 500 µM. The results are the mean of three independent experiments and the error bars represent the standard error (SE). In particular, effects of incubation time (peptide:liposomes) and of the lipid mixtures (different liposomes tested) on vesicle aggregation were statistically evaluated (**f**). The *p*-values were determined by two-way ANOVA analysis, performed using GraphPad Prism^®^ 8.0.1. ns means not significant; * *p* < 0.05; ** *p* < 0.01; **** *p* < 0.0001.

The mechanism of action of 1018-K6 was further investigated by exploring whether AMPs might interact simultaneously with one or more vesicles. Vesicle membrane surfaces may move closer together due to membrane-peptide-membrane interactions, leading to vesicle aggregation. Thus, in contrast to the typical dispersion of the vesicles in solution due to repulsive electrostatic forces, if 1018-K6 were capable of such simultaneous interactions, lipid vesicles would be expected to aggregate. Indeed, 1018-K6 did induce aggregation of all the model bacterial membrane vesicles, both Gram-negative and Gram-positive (Figure 9). Even at very low concentrations (Figure 9a), the difference between the zwitterionic and anionic vesicles was notable, and this significant difference (Figure 9e) was amplified as the peptide dose increased and as the C_L_/C_P_ ratio decreased. Furthermore, the peptide apparently preferred more negatively charged membranes than neutral ones, displaying a very high affinity for *S.* Typhimurium model MLVs. The binding of the cationic peptides to the membranes could disturb their electrostatic forces and affect the stability of the membrane surface [51], thereby dampening the repulsions between MLVs.

## 3. Discussion

Relevant information is provided here regarding the mechanism of action of the AMP 1018-K6, in particular enhancing the information available regarding the determinants of peptide–membrane interactions and their involvement in the role of 1018-K6 as a bactericidal and antibiofilm compound against *Listeria monocytogenes* and *Staphylococcus aureus* isolates, and strains of different *Salmonella* serotypes [25,28,29,30]. Indeed, binding experiments using MLVs from commercially available lipids that mimic cell membranes, and those with lipids extracted from biological membranes of *Salmonella* spp., *E. coli* and *S. aureus* (Figure 1 and Figure 2, and Table 1) confirmed the specific binding of 1018-K6 to bacterial cell membranes and not to eukaryotic plasma membranes. 

In this context, centrifugation binding studies showed 10- to 100-fold stronger binding of 1018-K6 to *Salmonella* and *Staphylococcus* model membranes than to eukaryotic membranes (B_max_ values) (Figure 1 and Figure 2, and Table 1). Absorbance and fluorescence spectroscopy analyses produced similar results, indicating that these approaches were appropriate to assess this phenomenon. In addition, measuring the distribution of 1018-K6 with the partition coefficient, *K_p_*, (Table 1) also indicates that the peptide’s binding capacity to bacterial membranes was up to 10-fold higher than that of model eukaryotic membranes. Moreover, binding to biological membranes replicated the data obtained in model membranes. Several conclusions can be drawn from these binding studies. First, that the use of model membranes formed with commercially available lipids in proportions that mimic natural bilayers gives similar results to those obtained with membranes formed from lipids extracted from biological membranes, further validating the models and results presented elsewhere [52]. Secondly, the 1018-K6 peptide binds strongly to bacterial membranes but not to eukaryotic membranes, specificity that, in part, explains the antibiotic potential of this peptide. Moreover, 1018-K6 interacts distinctly with membranes of different bacteria. Regarding the differences in binding to Gram-positive or Gram-negative bacteria, the results obtained using *E. coli* model membranes can be confusing, as the B_max_ values better resemble those of *S. aureus* model membranes than those of the other Gram-negative bacteria. However, a shift in the SDS content from 1 to 2 % when measuring these model membranes may explain this result. Difficulties in the spectroscopic measurements of the Trp residues of peptides in the *E. coli* model membrane, which required slight increases in SDS final concentration from 1 to 2 % to fully disrupt the membrane for Trp concentration determination, might be involved in the inconsistency of this set of data for this model membrane. It is known that phosphatidylethanolamine (the main constitutive lipid of this model membrane) isolated from several sources tends to form hexagonal phases (H_II_) in vitro below physiological temperature, and that Boggs et al. [53] determined that the temperature of the lamellar to hexagonal (L-H) phase transition of the bovine white matter PE is 18 °C. Moreover, it has been demonstrated that short peptides are capable of inducing a reversed hexagonal (H_II_) phase and, to this purpose, Morein et al. [54] have studied the effect of hydrophobic peptides at several lengths on the lipid phase behavior of a model membrane of *E. coli* (dioleoylphos-phatidylethanolamine (DOPE)/ dioleoylphosphatidylglycerol (DOPG), 7:3 molar ratio). Therefore, since many of the conditions described above occur in the experimental tests of the present study (the incubation temperature close to 18 °C and use of a short peptide), it can be hypothesized that the recorded B_max_ and *K_d_* values measured for *E. coli* model membranes are influenced by the PE lipid transition to the hexagonal phase.

In summary, this series of experiments indicate that there could be a huge difference in 1018-K6 binding to human or bacterial membranes, expressing some specificity for certain bacteria. These results are based upon specific peptide-lipid interactions and support the development of different lipids to combat different bacteria. Membrane lipid therapy (MLT, melitherapy) involves using the plasma membrane as a target for different interventions [4,55,56]. The relevant differences between human and bacterial membranes pave the way to develop melitherapy compounds that alter the membrane of specific bacterial pathogens while not affecting human membranes. These interactions aim to kill pathogenic bacteria so as to preserve food from an infection that could threaten the consumer’s life, or to treat patients infected with bacteria resistant to other antibiotics. The results obtained here are encouraging and suggest this approach is feasible.

The fluorescence properties of 1018-K6 were used to define the location of the peptide and its interactions with bacterial membranes (Figure 3, Figure 4, Figure 5, Figure 6 and Figure 7, Table 2, Table 3, Table 4 and Table 5). A blue shift in emission spectra (Figure 3) suggests integration into the membrane, as further confirmed in fluorescence quenching experiments with acrylamide (Figure 4 and Figure 5, Table 2 and Table 3) and nitromethane (Figure 6 and Figure 7, Table 4 and Table 5). The blue-shift experiments (Figure 3) indicate that the interaction of 1018-K6 with *Staphylococcus* membranes differed from other interactions and the huge shift of the emission maximum of Trp in these membranes indicates that the peptide was in a highly hydrophobic environment, a situation limited to the acyl chains of membrane phospholipids. By contrast, peptides may also insert fully into membranes, with the Trp residue situated close to the interfacial region of the bilayer polar head or the inter-monolayer space, which also contains the water molecules that give this membrane region significant polarity.

Fluorescence quenching experiments were also performed (Figure 4, Figure 5, Figure 6 and Figure 7, Table 2, Table 3, Table 4 and Table 5), first using acrylamide as a water-soluble quenching agent to determine with which membranes 1018-K6 established shallow or deep membrane interactions. In this context, the quenching of 1018-K6 Trp fluorescence in solution (buffer) (Figure 4b and Figure 5a) was very similar to that of eukaryotic model membranes, indicating that the peptide does not insert into eukaryotic membranes. Thus, 1018-K6 should not affect human membranes, as further supported elsewhere in this study. By contrast, acrylamide did not cause significant quenching of Trp fluorescence in bacterial membranes (Figure 4e,f and Figure 5b–d), indicating that this hydrophilic quencher could not access the 1018-K6 peptide as it was most likely inserted into the bacterial membrane. In this context, the *K_SV_* value for acrylamide was one order of magnitude higher for eukaryotic model membrane than for *Salmonella* or *Staphylococcus* and, moreover, in model *Staphylococcus* membranes there was a 10-fold time-dependent reduction in the *K_SV_* value over 150 min (Table 2 and Table 3). This effect, which was not observed for membranes resembling *Salmonella* (Figure 4e)*,* indicates that the binding and internalization kinetics of 1018-K6 differed in these membranes, additional evidence of the specificity of AMPs for different bacteria. Furthermore, the slow kinetics in *Staphylococcus* membranes (Figure 4f and Figure 5d)*,* suggests that several units of the 1018-K6 peptide could assemble as oligomers, a result that merits further study.

The fact that both a zwitterionic membrane formed with the main bulk phospholipid found in eukaryotic membranes (PC) (Figure 4c) and a model eukaryotic membrane (PC_40_:POPE_40_:SM_15_:PS_5_) (Figure 4d) had a similar *K_SV_* to that of 1018-K6 in buffer (Figure 4a,b, Table 2) clearly indicates that the interaction of this peptide with human membranes was weak, and that it occurred at the surface of the membrane. Hence, the interaction of this AMP with eukaryotic membranes is very mild and there is no effect on the integrity of these bilayers (see below), further demonstrating that 1018-K6 should not affect human cell membranes. This poor binding to eukaryotic membranes suggests that 1018-K6 could be used to preserve food and to combat bacterial infections in cases of resistance against available antibiotics. Finally, the *K_SV_* values measured in lipids obtained from bacteria (*Salmonella* spp., *Staphylococcus aureus* and *Escherichia coli*) are almost identical to those results obtained in model membranes established with commercially available lipids. Hence, model membranes appear to contribute reliable data regarding the location of the 1018-K6 peptide in the lipid bilayer.

Nitromethane is a polar/apolar quencher that can insert into membranes [57,58] and thus, this amphipathic molecule can quench fluorophores in both the aqueous phase and in membranes. Therefore, the difference between the *K_SV_* values for nitromethane quenching of 1018-K6 Trp fluorescent emission in eukaryotic and prokaryotic membranes was similar (107 M^−1^, 55 M^−1^ and 55 M^−1^ for eukaryotic, *Salmonella* and *Staphylococcus* model membranes, respectively, Table 3). This similarity in *K_SV_* values contrasts with the quenching obtained with acrylamide, where the *K_SV_* value for the model eukaryotic membranes was ca. 2600% that observed for *Salmonella* membranes (Table 2). Moreover, the *K_SV_* value in *Staphylococcus* membranes increased to 4-fold that observed in *Salmonella* model membranes after 30 min (Table 3). Together, the quenching of 1018-K6 Trp fluorescence observed with acrylamide and nitromethane clearly indicates that this peptide inserts into bacterial membranes, but it interacts poorly with eukaryotic membranes. This selectivity is a basic aspect required for antibiotic effectivity, and it supports the use of the differences between eukaryotic and prokaryotic plasma membranes to develop new antibiotics capable of eluding the conventional mechanisms of resistance developed by bacteria.

The efflux kinetics of a solute encapsulated in a lipid vesicle has been widely used to study the activity of AMPs [59]. Thus, this type of experiment detects whether pore opening is maintained and stable or if by contrast, the pores that form are transient and unstable. CF leakage from unilamellar membranes was used to study the mechanism of action of 1018-K6 and in these vesicles, external CF molecules were eliminated from the medium by exclusion size chromatography (see Materials and Methods). CF is a low molecular-weight, hydrophilic and polar molecule widely used to study the tissue distribution of liposomes [60,61], and it is retained by the lipid bilayer unless the latter is altered by discontinuities induced through different factors [62]. The high concentration of CF inside vesicles induces self-quenching, whereas the release of this molecule into the medium is associated with increased fluorescent emission. In this scenario, membranes from bacteria were associated with an increase in fluorescence up to 90% of the maximum fluorescence (Figure 8), as determined upon total membrane disruption by detergent treatment. By contrast, the presence of 1018-K6 was associated with modest CF release in zwitterionic (PC) and eukaryotic cell membrane (PC_40_:POPE_40_:SM_15_:PS_5_). These experiments suggest that CF could induce pores at least 1 nm in diameter in bacterial membranes, pores that would alter the integrity of the bacterial cell, which in turn might release its content. This bacterial life-threatening process could underlie the activity of various AMPs against bacteria and represent an elegant strategy to develop new therapies to fight bacterial infection.

Some hypotheses have been formulated regarding the potential of 1018-K6 to fragment bacterial membranes [25,28]. The antibiofilm activity of the peptide was demonstrated by scanning electron microscopy (SEM), providing more information on its potential mechanism of action. Indeed, treating the preformed biofilm of *S. aureus* and *Listeria monocytogenes* with 1018-K6 at 80 and 50 μM, respectively, largely eradicates the biofilm within 16 h, with signs of cell membrane damage and blebbing of the membrane surface. The experimental protocol used here could not define any possible effect of the peptide on the membrane of bacterial cells, although the difference in the incubation times (only 30 min in the binding studies) could explain the discordance in these results.

For many years the scientific community has been aware of how numerous physicochemical features of AMPs define their general activities. Charge, length, amino acid composition, hydrophobicity and amphipathicity are some of the main features driving the activity of AMPs [63,64]. The binding affinity to Gram-positive membranes of an AMP with an arginine (Arg-R) in its amino acid sequence pointed to an important role of the guanidinium side chain in the binding process [65]. This may be due to the strong ability of the guanidinium group to create a solid bidentate H-bond with phosphate moieties or cation–p interactions with aromatic residues [66]. This hypothesis was based on the differences observed between the model membranes of Gram-positive and Gram-negative bacteria. In particular, when the binding properties of three peptides with different amino acid sequences were compared, the changes in sequence did not affect the affinity of peptides for Gram-negative membranes. However, the replacement of lysine (Lys-K) with Arg significantly enhanced their affinity for Gram-positive bacteria, marking the difference in the activity of the peptides. Moreover, the addition of Trp to the peptide sequence confers greater affinity for lipid membranes [67], improving its insertion into the lipid core by inducing the penetration of Arg through cation–p interactions [68].

The first barrier that AMPs encounter when in contact with bacteria is the cell wall. For this interaction, two features are critical: the net charge and the hydrophobicity of the AMPs. CAMPs can interact electrostatically with negatively charged cell wall components, such as lipopolysaccharide (LPS), for Gram-negative bacteria, and lipoteichoic acid (LTA), for Gram-positive bacteria. This initial interaction of AMPs with LPS can lead to membrane destabilization and permeabilization [69,70,71,72]. Although several authors have already demonstrated the ability of cationic peptides to interact with the polyanionic surface of LPS and LTA by competitively supplanting the divalent cations that link and partially neutralize the outer membrane or cell wall, in our previous study we have collected useful information that has revealed the potential ability of 1018 -K6 to interact with them. The images obtained by SEM and TEM showed how the peptide is able to alter the bacterial membrane, visible as a surface swelling, after going through the cell wall (LTA) of *Staphylococcus aureus* and *Listeria monocytogenes*, respectively [26,28]. This result was surprising and encouraging, being aware of the opinion of the scientific community that considers LTA as a more complicated challenge than LPS for cationic peptides [71]. Hydrophobic residues, such as tryptophan, then allow the AMPs to enter the bacterial membrane bilayer.

There are several models of antimicrobial peptide activity [68,73,74]. Three of them start from the same conformation, with the peptides associating with the bacterial membrane as the barrel-stave model, the carpet model (also causing pore formation), and the model of toroidal pore, which creates pores in which peptides and lipids are intermingled and bend into the pore continuously from the surface of the membrane. Other models include the molecular electroporation model based on an electrical potential generated by CAMPs [75,76] and the sinking raft model based on a mass imbalance that generates a local curvature of the membrane. Given the heterogeneity of structures and sizes of AMPs, each peptide could use one or more of these models in its interactions with membranes.

Several AMPs also exhibit lipid vesicle fusogenic activity [77], so we performed vesicle aggregation studies with our peptide to obtain additional information on its mechanism of action. As 1018-K6 promotes vesicle accumulation, it is one of many peptides that have both mixed pore-forming and membrane fusogenic activities. Thus, this dual effect would confer the 1018-K6 AMP an additional advantage in terms of its lytic effect that may be propagated in bacterial populations.

All in all, we show that 1018-K6 binds preferentially to bacterial cell membranes as opposed to eukaryotic plasma membranes and that this binding displays some species specificity. When 1018-K6 binds to bacterial membranes, it can disrupt the integrity of the target cell’s membrane, an effect that propagates to neighboring cells via aggregation. Although future imaging studies and a better characterization of the pore opening in the bacterial membrane could provide further information, the data presented here helps understand the lytic activity of 1018-K6 on bacterial populations.

## 4. Materials and Methods

### 4.1. Materials

1-palmitoyl-2-oleoyl-sn-glycero-3-phosphoethanolamine (POPE), L-α-lysophosphatidylcholine (Egg Lecithin, PC), L-α-phosphatidylethanolamine (Liber Bovine, PE), 1′,3′-bis [1,2-dioleoyl-sn-glycero-3-phospho]-glycerol (Cardiolipin 18:1, CL), phosphatidyl serine (PS), and 1,2-dioleoyl-sn-glycero-3-phosphoethanolamine (DOPE) were purchased from Avanti Polar Lipids (Alabaster, AL, USA), while 1-palmitoyl-2-oleoyl-*sn*-glycero-3-phosphatidylglycerol (POPG) and N-Acyl-D-sphingosine-1-phosphocholine (chicken egg yolk, SM) were provided by Larodan AB (Solna, Sweden) and Sigma-Aldrich (St. Louis, MO, USA), respectively. Tris-HCl, Tris base, Sephadex G-100, 5-carboxyfluorescein (≥95%, HPLC), nitromethane (Reagent Plus, ≥99%), Triton™ X-100 and acrylamide were purchased from Sigma-Aldrich (St. Louis, MO, USA). Sodium Dodecyl Sulfate (SDS, 20% Solution) and Luria Broth (LB) were purchased from Thermo Fisher (Bremen, Germany). Ammonium heptamolybdate tetrahydrate ((NH_4_)Mo_7_O_24_·4H_2_O), di-sodium hydrogen phosphate anhydrous (Na_2_HPO_4_), L(+)-Ascorbic acid and all the other reagents were obtained from Scharlau (Barcelona, Spain). The derivative 12-mer peptide 1018-K6 was purchased from SynPeptide Co., Ltd. (Shanghai, China).

### 4.2. Preparation of Model Membranes: Multilamellar Vesicles (MLVs)

Model membranes (liposomes) were made using lipid stock solutions of POPE, PE, DOPE, PC, SM, PS, CL and POPG (10 mM), obtained by dissolving the lipid in chloroform:methanol (2:1, v/v). Lipid films were prepared in glass tubes by mixing an appropriate volume of phospholipids to achieve the desired molar ratio (Table 6). Subsequently, the solutions were dried under argon (Ar) flow and then subjected to a vacuum for at least 3 h to remove traces of the solvent. A sufficient volume of binding buffer (Tris HCl 50 mM, NaCl 100 mM pH 7.4) was used to resuspend the lipidic film, yielding 2 mM lipid phosphorus. Fresh lipid suspensions were prepared before each experiment.

### 4.3. Preparation of Biological Membranes from Bacterial Strains and Hepatic Rat Tissue

Wild strains of *Staphylococcus aureus*, *Escherichia coli* and *Salmonella* spp. were isolated from foods and used to evaluate the binding affinity of 1018-K6 to bacterial membrane lipids. These bacteria belong to the Type Culture Collection of the Laboratory of Food Microbiology of the Department of Veterinary Medicine and Animal Production (Federico II, University of Naples). In particular, *S. aureus* was isolated from buffalo cheese, while *E. coli* and *Salmonella* spp. was obtained from wild boar carcass and other meats, respectively. Bacterial identification was performed by the “direct colony identification method” [80] and processed with a MALDI Biotyper^®^ sirius System (Bruker Daltonics, Bremen, Germany) [81]. Identification was achieved by comparing each bacterial mass spectra to the Bruker MSP database using Bruker Compass software (Bruker Daltonics). The bacterial cultures were then freeze-dried in 20% (v/v) glycerol and stored at −80 °C. Cultures of *S. aureus*, *Salmonella* spp. and *E. coli* were grown overnight at 37 °C in LB (formulation for 1 L: 10 g Select Peptone 140, 5 g Select Yeast Extract, and 5 g NaCl). When the desired cell concentration was reached (exponential phase), the bacterial culture was separated into 50 mL Falcon tubes and centrifuged (Sigma, 2-16 K, rotor 231/F; Osterode am Harz, Germany) at 8600× *g* (9000 rpm) for 10 min at 4 °C. The supernatant was then removed, and the pellet used for lipid extraction.

Eukaryotic plasma membranes were isolated from rat hepatic tissue (2g) homogenized (Homogenizer Polytron PT-310, Kinematica AG, Malters, Switzerland) with 38 mL of binding buffer supplemented with protease inhibitors (cOmplete™, EDTA-free Protease Inhibitor Cocktail; Roche, Mannheim, Germany) to a final 20-fold dilution. The homogenate was transferred to Eppendorf tubes and centrifuged (Thermo Scientific ST 8R, rotor 75005715, Waltham, MA, USA) at 1000× *g* for 15 min at 4 °C. The supernatant was then collected and transferred to clean Eppendorf tubes and centrifuged again (Thermo Scientific ST 8R, rotor 75005715) at 10,000× *g* for 15 min at 4 °C. At the end of the second centrifugation, the supernatant was collected into polycarbonate tubes (10 mL, 16 × 76 mm: Beckman Coulter, Pasadena, CA, USA) and centrifuged at ca. 50,000× *g* (30,000 rpm) for 1 h at 4 °C (Beckman LE-80 Ultracentrifuge; rotor type 70.1Ti).

The following lipid extraction steps were the same for both bacterial and eukaryotic membranes. Lipid extracts of biological membranes were obtained following the experimental protocol of Dennison et al. [82] with some modifications. Briefly, each resulting pellet was resuspended in 1 mL of Tris Buffer (25 mM, pH 7.5) and the final solution was transferred into a 10 mL glass tube, to which 3.57 mL of a 1:2 (*v*/*v*) chloroform:methanol mixture was added, and the cells were vigorously vortexed for 5 min. Then, 1.25 mL of chloroform and 1.25 mL of distilled water were added, vortexing for 5 min between each addition. After adding the water, the solution was again vortexed for 5 min and then centrifuged (Thermo Scientific ST 8R, rotor 75005701) at low speed (1000× *g*) for 10 min at 4 °C to obtain three separated phases: organic, aqueous and protein (bottom, upper and center phases, respectively). The organic phase was transferred into a clean 10 mL centrifuge tube and 4.75 mL of a 1:1 (*v*/*v*) methanol-buffer mixture was added, while 500 μL of chilled chloroform was added to the remaining aqueous phase. The two solutions were vortexed vigorously for 5 min, centrifuged as described above, and the respective organic phases were collected. Once the organic phases were unified, the solution obtained was dried under an Ar flow and then subjected to a strong vacuum for at least 3 h. The lipid films were resuspended in binding buffer (Tris HCl 50 mM/ NaCl 100 mM; pH = 7.4) and a Fiske assay was performed to determine the lipid phosphorus concentration (see Section 4.8).

All animal experiments were carried out in accordance with the animal welfare guidelines of the European Union (Directive 2010/63/EU) and the regional government of the Balearic Islands (Conselleria d’Agricultura, Pesca i Alimentació, code: 2018/09/AEXP, date of approval: 10 August 2018).

### 4.4. Lipid Binding Assay

To assess the extent of the peptide-MLV interactions, reaction mixtures were prepared in Eppendorf tubes combining each lipid solution (at the fixed molar concentration of 1800 µM) with pre-formed liposomes containing different amounts of peptide, ranging from 15.63 to 78.13 µM (when saturation did not occur, higher peptide concentrations were tested). The solutions were vortexed vigorously and incubated at room temperature for 30 min to allow binding. As negative controls, analogous samples were made using the buffer solution vehicle rather than the stock peptide solution. At the end of the incubation period, the solutions were carefully transferred to polycarbonate centrifuge tubes (1 mL, 8 × 51 mm: Beckman Coulter, USA) and centrifuged at ca. 60,000× *g* (20,000 rpm, outer row) for 1 h at 20 °C (Beckman LE-80 Ultracentrifuge; rotor type 25 [31]). After centrifugation, the supernatant was removed and mixed with binding buffer in the presence of SDS to a final concentration of 1–2%. The pellet was washed with binding buffer and resuspended in the same buffer containing SDS. The binding of the peptide to the model and biological membranes was assessed by quantifying the amount of 1018-K6 in the pellet and supernatant using calibration curves generated by adding known amounts of peptide to control supernatants or pellets of vesicles prepared in the absence of the peptide. Peptide binding to multilamellar vesicles (MLVs) was measured by absorbance and fluorescence spectroscopy of the tryptophan residue in 1018-K6.

#### 4.4.1. Steady-State Tryptophan Fluorescence

Tryptophan (Trp) fluorescence spectra were recorded for each supernatant and pellet sample after 30 min of stirring at 900 rpm in a Thermo-Shaker (TS-100: Biosan) at room temperature. The variation in Trp emission was recorded between 300 and 450 nm excitation, at λex = 280 nm, using a Cary Eclipse Fluorescence spectrophotometer (Varian, Sydney, Australia). Slit widths were 5 nm for both excitation and emission, and each spectrum was corrected by subtracting the liposome background.

#### 4.4.2. UV-Vis Absorption Spectrophotometry

Peptide quantification was also performed by UV absorbance of the Trp residues at 280 nm using a UV-Vis Cary 300 Bio Spectrophotometer (Varian, Australia). To this end, the samples were treated as described above for fluorescence measurements and a saturation binding analysis was performed with GraphPad Prism^®^ using the Scatchard analysis, estimating the *K_d_* (equilibrium dissociation constant) and B_max_ (maximum specific binding) values (Equation (1)). B_max_ is the total number of binding sites and it is expressed in the same units as the *y* values (nmol/mg lipid), while the *K_d_* represents the concentration of ligand, which at equilibrium occupies 50% of the receptors present in the biological preparation. It is expressed in the same units as the ligand (peptide, P), [P]*_total_* (µM).

(1) $${\left[P\right]}_{bound}=\frac{B_{max}\times {\left[P\right]}_{total}}{K_{d}+{\left[P\right]}_{total}}$$

Plotting the ratio between the quantity of bound and free ligand ([P]*_bound_*/[P]*_free_*) with respect to the amount of bound ligand ([P]*_bound_*) gives a straight line with a slope of −1/*K_d_*. The intercept on the abscissa axis (([P]*_bound_*/[P]*_free_* = 0) defines the receptor density (B_max_). In other words, this analysis allows the peptide binding affinity and the density of binding sites for these peptides in lipid bilayers to be determined.

Furthermore, the partition coefficient (the equilibrium distribution of 1018-K6 between the membrane and the solution), *K_p_*, was calculated by fitting the experimental data with Equation (2), as described by White et al. [83]. This equation considers the peptide distribution between hydrophobic membranes in the pellet (membrane-bound peptide) and the hydrophilic supernatant (free peptide) upon centrifugation [84]. For this reason, considering that the amount of peptide bound is the result of subtracting the free peptide from the total amount of peptide, the partition coefficient can be calculated as:

(2) $$K_{p}=\frac{\frac{{\left[P\right]}_{total}-{\left[P\right]}_{free}}{\left[L\right]}}{\frac{{\left[P\right]}_{free}}{\left[W\right]}}$$

where [P]*_total_* and [P]*_free_* are the aqueous peptide concentrations measured before the addition of the vesicles and after centrifugation, respectively; and [L] and [W] are the molar lipid concentrations (55.3 M). It must be noted that the model membranes were preformed before adding the peptide, which prevents 1018-K6 precipitation within liposomes in the absence of peptide-membrane interactions.

The values of binding potential (BP = B_max_/*K_d_*) were also calculated as described elsewhere [85,86].

Bacterial lipid extracts were used to prepare model membranes to assess peptide binding to biological lipid bilayers. This experimental design allowed the binding of 1018-K6 to real bacterial membranes to be assessed. The reaction mixtures were prepared as described above for model membranes (MLVs) from synthetic lipids. Thus, lipid solutions at fixed lipid phosphorus concentrations (µg/µL) were incubated with different amounts of peptide. Due to the low lipid extraction yield from bacterial cultures, peptide binding was analyzed to maintain the same lipid:peptide molar ratio. Thus, the ratio between the lipid phosphorus concentration (C_L_, µg/µL) for model membranes prepared from *S. aureus*, *S. enterica* or *E. coli* lipids, and the peptide concentration (C_P_, µg/µL) were the same as those used for liposomes formed with synthetic lipids. This approach allowed binding results from biological membranes to be compared with those of model synthetic membranes resembling bacterial and human membranes. Spectroscopic Trp measurements (absorption and fluorescence) were determined for each sample of supernatant and pellet as described above. The B_max_ and *K_d_* values were calculated from a Scatchard analysis of the data using GraphPad Prism^®^ version 8.0.1 (GraphPad, San Diego, CA, USA).

### 4.5. Vesicle Aggregation

The ability of the AMP to alter the lipid bilayer surface and induce vesicle aggregation was followed by optical density measurements at 436 nm (OD_436_ [87]). Different concentrations of the peptide (6.6, 12.5, 20, 25 and 50 µM) were incubated with MLVs at 500 µM. This assay was performed in flat-bottom 96-well plates and the turbidity variations were followed over the first 35 min of the peptide-lipid incubation using a FLUOstar Omega spectrophotometer (BMG LABTECH, Ortenberg, Germany).

### 4.6. Absorption Maximum and Quenching of 1018-K6 Fluorescence in Aqueous and Lipid Environments

Membrane-peptide interactions were also investigated by analyzing the blue shift in the Trp emission spectra. In particular, insertion of lipophilic fluorophores into hydrophobic regions of the lipid bilayer induce a blue shift of the emission maximum. To investigate membrane-peptide interactions, a fixed amount of the peptide 1018-K6 (20 μM) was incubated for 30 min at room temperature in the presence or absence of increasing concentrations of lipids (final lipid phosphorus concentration in the vesicle suspension ranging from 0–2 mM). Fluorescence was measured in 1 cm path length quartz cuvettes on a Cary Eclipse Fluorescence spectrophotometer (Varian, Australia). The Trp emission intensity and wavelength were recorded between 305 and 490 nm, and the integrated area of each fluorescence spectrum was corrected by subtracting liposome scattering.

In addition, the exposure of the Trp residue in 1018-K6 to the aqueous environment was evaluated by fluorescence emission quenching upon titration with acrylamide and nitromethane. Fluorescence quenching was determined using the Stern–Volmer equation and the Stern–Volmer constants (*K_sv_*) were calculated as described elsewhere [47]. Five (5) µL aliquots of a 4.0 M stock of the quenching solution (acrylamide or nitromethane) were added to the cuvette containing samples of phospholipid forming MLVs (30 µM) and peptide (1 µM) in a total volume of 1 mL. The *K_sv_* values were calculated from the equation (Equation (3)):

F_0_/F = 1 + *K_sv_*(Q) (3)

where F_0_ is the initial fluorescence of the peptide and F is the fluorescence intensity following the addition of different concentrations of the quencher, Q.

### 4.7. Permeabilization of Model and Biological Membranes

Lipid films from model (PC, PC_40_:POPE_40_:SM_15_:PS_5_, CL_42_:POPG_58_, and DOPE_78_:POPG_18_:CL_4_) and biological membranes (*S. aureus* and *Salmonella* spp.) were prepared as described above. The lipid films were then resuspended in binding buffer containing 5-carboxyfluorescein (CF, 25 mM) by vigorous vortexing to obtain MLVs at a final concentration of 5 mM in lipid phosphorus. Large unilamellar vesicles (LUVs) were prepared by extrusion of MLVs by 11 passages through 1 µm pore-size polycarbonate membranes in a miniextruder (Avanti Polar Lipids, Inc., Alabaster, AL, USA), after six freeze-thaw cycles. Free CF not incorporated into the vesicles was removed by exclusion chromatography of the liposome suspension (200 µL) through a Sephadex G-100 column (10 cm long and 1 cm in diameter). CF-loaded LUVs were collected in glass vials and their lipid phosphorus concentration was determined by carrying out the Fiske test (see Section 4.8 below). LUV permeabilization kinetics were followed by detecting the increase in CF fluorescence emission (λ*ex* = 490 nm, λ*em* = 517 nm; Cary Eclipse Fluorescence spectrophotometer: Varian, Australia) following release from the vesicles and the ensuing loss of CF self-quenching. In these experiments, a fixed concentration of LUVs (10 µg/mL of lipid phosphorus, about 125 µM) was incubated with the 1018-K6 peptide (15 µM). To determine the CF loaded in LUVs, 0.25 % Triton X-100 was added to the cuvette after 30 min of peptide-LUV incubation and the CF release that corresponded to that of permeabilization, was calculated as described [7]:

Permeabilization (%) = (*F_Pep_* − *F_N_*)/(*F_P_* − *F_N_*) × 100 (4)

where *F_N_* and *F_Pep_* corresponds to the fluorescence intensity after the addition of the peptide and prior to adding the peptide, respectively, while *F_P_* is the maximum fluorescence intensity after 100% CF externalization. The experiments were performed at 20 °C.

### 4.8. Lipid Sedimentation Assay

The ability of 1018-K6 to disrupt membranes was evaluated by detecting the presence of membrane fragments (smaller bilayer structures) in the supernatant after centrifugation. Thus, the lipid phosphorus concentration (1800 µM initial lipid phosphorus) was assessed in the pellets and supernatants after centrifugation. According to the protocol used to study the binding affinity of the peptide to liposomes (see Section 4.4), reaction mixtures were prepared by adding a specific amount of peptide (the same concentrations as used for the binding assay) to each lipid solution (1800 µM). After a 30 min incubation, the reactions were centrifuged at ca. 60,000× *g* for 1 h at 20 °C (Beckman LE-80 Ultracentrifuge; rotor type 25, outer row) and the lipid phosphorus concentration of MLVs in the pellets and supernatants was then determined using the Fiske assay [88,89,90] with slight modifications. Briefly, a calibration curve with different volumes of 1 mM Na_2_HPO_4_ was generated for each experiment, with 30 µL of each sample placed in a glass tube, and then 500 µL of 70% perchloric acid was added to each tube. After vortexing, the tubes were incubated at 180 °C for 45 min in a thermoblock (Multiplaces, Selecta, Cham, Switzerland) and at the end of the incubation period, the tubes were allowed to cool and 4 mL of molybdate ((NH_4_)Mo_7_O_24_·4H_2_O 1.89 mM and 14.3 mL of H_2_SO_4_ at 95–97%, diluted to 1 L with purified water) and 500 µL of 10% ascorbic acid (*w*/*v*) were added to each tube. Vortexed samples were incubated in boiling water for 5 min, and when the tubes cooled down, 250 µL of each sample and standard were transferred to 96-well plates to read the absorbance using a UV Spectrophotometer (FLUOstar Omega, BMG LABTECH, Offenburg, Germany). The optical density was determined at 800 nm, and the standard’s ODs were plotted against the known lipid concentration and used as a calibration curve to determine the lipid concentrations in the samples.

### 4.9. Statistical Analyses

Statistical analyses were performed using the SPSS software package (version 26, IBM Analytics, Armonk, NY, USA) and GraphPad Prism^®^, version 8.0.1 (GraphPad, San Diego, CA, USA). All experiments were performed at least three times and the data were presented as the mean (M) ± standard error (SE). GraphPad Prism^®^ was used to perform Scatchard analyses, student’s t-test (*p* < 0.05) and two-way ANOVA to assess the data from the peptide binding analyses, the peptide fluorescence assays (blue shift and quenching assays) and the vesicle aggregation, respectively. Partition constant values were analyzed using SPSS with generalized linear mixed models (GLMMs) and the means were compared using the Tukey test (*p* < 0.05).

## 5. Patents

International Patent, Application No. PCT/EP2018/069304Publication Date: 16 July 2018. Antimicrobial peptides. Italian Patent, Application No. 102017000080068 Publication Date: 14 July 2017. “Peptidi antimicrobici”. Balestrieri M., Palmieri G., Neglia G., Anastasio A., Capuano F., de Stefano L., Nicolais L. Granting date 11 October 2019.

## Figures and Tables

**Table 1:** Partition constants for AMP 1018-K6 in multilamellar vesicles of model membranes.

Cell Type	Lipid Mixture	*K_p_*/(×10^4^ M^−1^)	
		Fluorescence	UV-Vis Absorption
Zwitterionic membrane	PC	0.76 ± 0.21 ^A^	0.83 ± 0.22 ^A^
Eukaryotic membrane	PC_40_:POPE_40_:SM_15_:PS_5_	0.37 ± 0.27 ^A^	0.10 ± 0.10 ^B^
*Salmonella* Typhimurium	DOPE_78_: POPG_18_:CL_4_	30.07 ± 4.53 ^B^	26.23 ± 4.87 ^C^
*Escherichia coli*	PE_80_:CL_5_:POPG_15_	36.50 ± 4.67 ^B^	14.65 ± 1.68 ^C^
*Staphylococcus aureus*	CL_42_:POPG_58_	19.69 ± 2.90 ^C^	16.11 ± 2.88 ^C^

The data was analyzed using SPSS version 26 (IBM Analytics, Armonk, NY, USA) and the results were analyzed with generalized linear mixed models (GLMMs), comparing the means using the Tukey test. All data were presented as the mean (M) ± standard error (SE): ^A^, ^B^, ^C^ values that differ at *p* < 0.01.

**Table 2:** Emission fluorescence quenching of 1018-K6 induced by acrylamide in the presence or absence of MLVs from model membranes.

Peptide: Liposome Incubation (mins)	Cell Type	Lipid Mixture	*K_SV_,* Acrylamide M^−1^	Equation	R Squared
30, 90, 150	Zwitterionic membrane	PC	154.8 ± 8.8 ^bAB^	Y = 154.80*X − 0.5730	0.9504
30, 90, 150	Eukaryotic membrane	PC_40_:POPE_40_:SM_15_:PS_5_	374.0 ± 47.9 ^acA^	Y = 374.00*X − 3.1910	0.7925
30, 90, 150	*Salmonella* Typhimurium	DOPE_78_: POPG_18_:CL_4_	14.4 ± 1.0 ^cC^	Y = 14.39*X + 0.9384	0.9333
30, 90, 150	Staphylococcus aureus *	CL_42_:POPG_58_	55.7 ± 21.2 ^acBC^	Y = 55.65*X − 0.3668	0.3020
30, 90, 150	Buffer	-	142.7 ± 16.3 ^a^	Y = 142.70*X − 1.1240	0.8279
30	* Staphylococcus aureus	CL_42_:POPG_58_	132.7 ± 39.1	Y = 132.70*X − 0.6068	0.4183
90	*Staphylococcus aureus*	CL_42_:POPG_58_	21.5 ± 6.1	Y = 21.46*X + 0.8326	0.4347
150	*Staphylococcus aureus*	CL_42_:POPG_58_	12.8 ± 1.0	Y = 12.81*X + 0.8746	0.9089

The *K_SV_* were calculated by fitting the results of three independent scans after 30, 90 and 150 min peptide:liposome incubations to the Stern–Volmer equation. Details of the 1018-K6 fluorescence quenching induced by acrylamide in the presence of *S. aureus* model membranes after 30, 90 and 150 min in the presence of the peptide. The data were analyzed with GraphPad Prism^®^ version 8.0.1 (GraphPad, San Diego, CA, USA), and the results were assessed by two-way ANOVA and a Tukey’s multiple comparison test. All the data were presented as the mean (M) ± standard error (SE): ^A^, ^B^, ^C^ values within the column differ at *p* < 0.01; ^a^, ^b^, ^c^ values in the columns differ at *p* < 0.05.

**Table 3:** Emission fluorescence quenching of 1018-K6 induced by acrylamide in the absence or presence of several MLVs of biological membranes.

Peptide:Liposome Incubation (mins)	Biological Membranes	*K_SV_*, Acrylamide M^−1^	Equation	R Squared
30, 90, 150	*Salmonella* spp.	11.0 ± 0.5 ^A^	Y = 11.01*X + 0.9797	0.9695
30, 90, 150	*Escherichia coli*	15.2 ± 0.4 ^A^	Y = 15.16*X + 1.001	0.9885
30, 90, 150	Staphylococcus aureus *	16.3 ± 4.6 ^A^	Y = 16.25*X + 0.9782	0.2666
30, 90, 150	Buffer	142.7 ± 16.3 ^B^	Y = 142.70*X − 1.1240	0.8279
30	* Staphylococcus aureus	23.3 ± 8.5	Y = 23.33*X + 0.9695	0.3218
90–150	*Staphylococcus aureus*	9.2 ± 0.3	Y = 9.17*X + 0.9869	0.9854

The *K_SV_* were calculated by fitting the results of three independent scans to the Stern–Volmer equation after 30, 90 or 150 min of the peptide:liposome incubation. Detail of 1018-K6 fluorescence quenching induced by acrylamide in the presence of *S. aureus* model membranes at 30, 90 and 150 min of the peptide-lipid incubation. The data were analyzed with GraphPad Prism^®^, as outlined previously, and the results were assessed by a two-way ANOVA and Tukey’s multiple comparison test. All data were presented as the mean (M) ± standard error (SE). ^A^, ^B^ values in the column differ at *p* < 0.01.

**Table 4:** Emission fluorescence quenching by nitromethane of 1018-K6 induced in the presence or absence of MLVs from model membranes.

Peptide:Liposome Incubation (mi)	Cell Type	Lipid Mixture	*K_SV_*, Nitromethane M^−1^	Equation	R squared
30, 90, 150	Zwitterionic membrane	PC	130.5 ± 15.8 ^A^	Y = 130.50*X + 0.1678	0.7817
30, 90, 150	Eukaryotic membrane	PC_40_:POPE_40_:SM_15_:PS_5_	107.5 ± 21.0 ^A^	Y = 107.50*X + 2.0950	0.5808
30, 90, 150	*Salmonella* Typhimurium	DOPE_78_: POPG_18_:CL_4_	54.7 ± 5.0 ^aB^	Y = 54.73*X + 0.8185	0.8626
30, 90, 150	*Staphylococcus aureus*	CL_42_:POPG_58_	54.9 ± 3.2 ^aB^	Y = 54.91*X + 0.5856	0.9384
30, 90, 150	Buffer	-	117.0 ± 11.0 ^b^	Y = 117.00*X − 0.3541	0.8564

The *K_SV_* were calculated by fitting the results of three independent scans (after 30, 90 and 150 min incubations with peptide:liposome) to the Stern–Volmer equation. Detail of the emission fluorescence quenching of 1018-K6 induced by nitromethane in the presence of *S. aureus* model membranes after a 30, 90 and 150 min peptide-lipid incubation. The data were analyzed with GraphPad Prism^®^, as outlined previously, and the results were assessed using a two-way ANOVA and Tukey’s multiple comparison test. All the data were presented as the mean (M) ± standard error (SE): ^A^, ^B^ values in the column differ at *p* < 0.01; ^a^, ^b^ values in the columns differ at *p* < 0.05.

**Table 5:** Nitromethane quenching of 1018-K6 fluorescence emission in the presence or absence of MLVs from several biological membranes.

Peptide:Liposome Incubation (mins)	Biological Membranes	*K_SV_*, Nitromethane M^−1^	Equation	R Squared
30, 90, 150	*Salmonella* spp.	50.6 ± 3.1 ^aA^	Y = 50.59*X + 0.7656	0.9328
30, 90, 150	*Escherichia coli*	9.0 ± 2.0 ^B^	Y = 8.95*X + 0.9698	0.5179
30, 90, 150	*Staphylococcus aureus*	55.3 ± 5.0 ^aA^	Y = 55.25*X + 0.4668	0.8650
30, 90, 150	Buffer	117.0 ± 11.0 ^bA^	Y = 117.00*X − 0.3541	0.8564

The *K_SV_* were calculated by fitting the results of three independent scans (after a 30, 90 and 150 min peptide:liposome incubation) to the Stern–Volmer equation. The data was analyzed with GraphPad Prism^®^, as outlined previously, and the results were assessed by two-way ANOVA and Tukey’s multiple comparison test. All the data were presented as the mean (M) ± standard error (SE): ^A, B^ values in the column differ at *p* < 0.01; ^a, b^ values in the columns differ at *p* < 0.05.

**Table 6:** Membrane lipid composition in eukaryotic and prokaryotic organisms (% mol).

Membrane	PC	SM	PS	PE	DOPE	POPE	POPG	CL
Eukaryotic membrane ^1^	40	15	5			40		
Zwitterionic membrane	100							
*Salmonella* Typhimurium ^2GN^					78		18	4
*Escherichia coli* ^3GN^				80			15	5
*Staphylococcus aureus* ^3GP^							58	42

^1^ [78]; ^2^ [79]; ^3^ [52]. PC, phosphatidylcholine; SM, sphingomyelin; PS, phosphatidylserine; PE, phosphatidylethanolamine; DOPE, dioleoyl-phosphatidylethanolamine; POPE, palmitoyl-oleoyl-phosphatidylethanolamine; POPG, palmitoyl-oleoyl-phosphatidylglycerol; CL, cardiolipin. GN, Gram-negative bacteria; GP, Gram-positive bacteria.

## Data Availability

Not applicable.

## Supplementary Materials

The supporting information can be downloaded at: https://www.mdpi.com/article/10.3390/ijms232012392/s1.
