# Supplementary material for: The Antimicrobial Peptide 1018-K6 Interacts Distinctly with Eukaryotic and Bacterial Membranes, the Basis of Its Specificity and Bactericidal Activity
Source: Int J Mol Sci. 2022 Oct 16;23(20):12392. doi: 10.3390/ijms232012392 (PMC9603936; doi:10.3390/ijms232012392)
Supplement: Supplementary file 1 [file ijms-23-12392-s001.zip › ijms-1950038-supplementary.pdf]

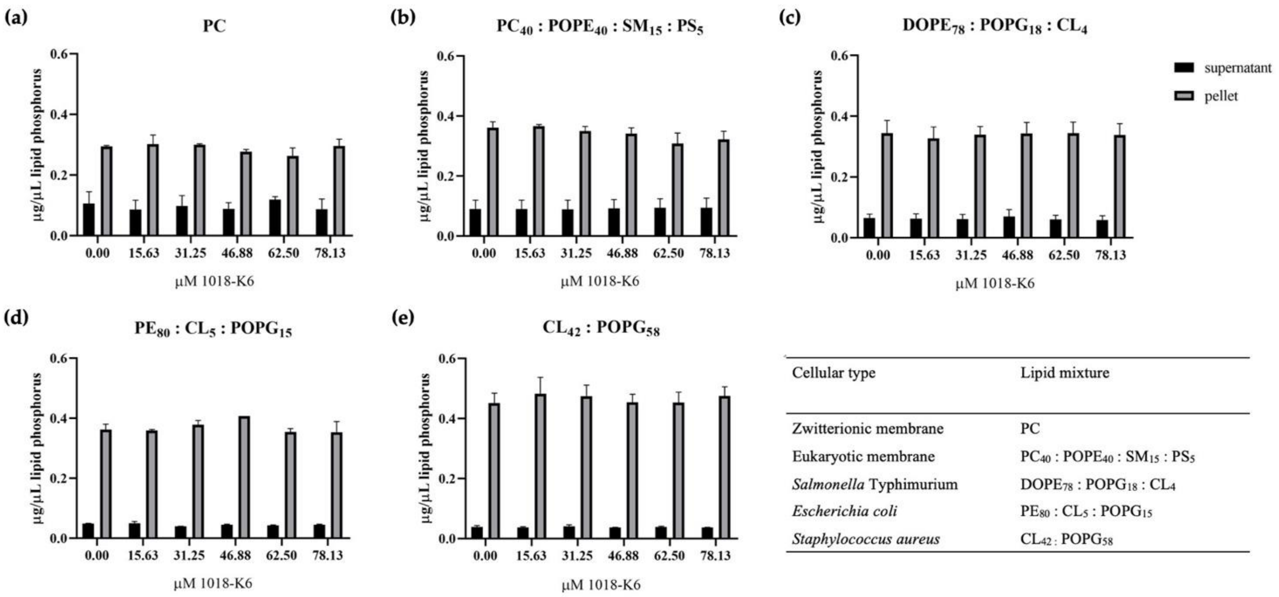

Cellular type	Lipid mixture
Zwitterionic membrane	PC
Eukaryotic membrane	PC_{40} : $POPE_{40}$: SM_{15} : PS_5
Salmonella Typhimurium	$DOPE_{78}$: $POPG_{18}$: CL_4
Escherichia coli	PE_{80} : CL_5 : $POPG_{15}$
Staphylococcus aureus	CL_{42} : $POPG_{58}$

Figure S1. Lipid phosphorus concentration in the supernatant and pellet for each multilamellar vesicle sample used to assess the binding to model membranes. Evaluation of the lipid sedimentation following a 30 minute incubation with several concentrations of the 1018-K6 peptide. **(a)** Zwitterionic lipids (PC); **(b)** Eukaryotic cell (PC_{40}:$POPE_{40}$:SM_{15}:PS_5); **(c)** *S.* Typhimurium ($DOPE_{78}$:$POPG_{18}$:CL_4); **(d)** *Escherichia coli* (PE_{80}:CL_5:$POPG_{15}$); **(e)** *S. aureus* (CL_{42}:$POPG_{58}$). The results correspond to the mean ± standard error (SE) of three independent experiments per model mem-brane.

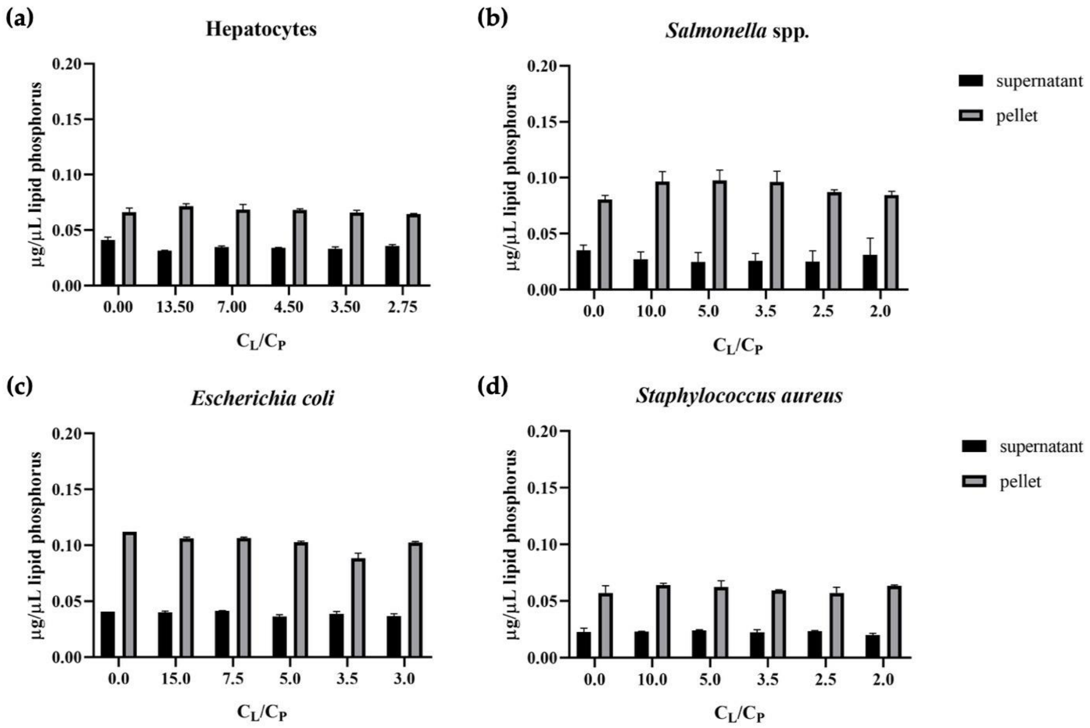

Figure S2. Lipid phosphorus concentration in the supernatant and pellet for each multilamellar vesicle sample used to assess the binding to biological membranes Evaluation of the lipid sedi-

mentation following a 30 minute incubation with several concentrations of the 1018-K6 peptide. (**a**) Hepatocytes; (**b**) *Salmonella* spp.; (**c**) *E. coli*; (**d**) *S. aureus*. The results correspond to the mean ± standard error (SE) of three independent experiments.

Table S1. Binding potential (*BP*) of AMP 1018-K6 towards multilamellar vesicles of model membranes.

Cell type	Lipid mixture	*BP*	
		Fluorescence	UV-Vis absorption
Zwitterionic membrane	PC	4.29	2.02
Eukaryotic membrane	$PC_{40} : POPE_{40} : SM_{15} : PS_5$	4.91	0.43
Salmonella Typhimurium*	$DOPE_{78} : POPG_{18} : CL_4$	5.17	3.37
Escherichia coli	$PE_{80} : CL_5 : POPG_{15}$	4.24	3.62
Staphylococcus aureus	$CL_{42} : POPG_{58}$	3.32	2.17

*The results refer to the experiments carried out with a higher concentration of peptide.

Table S2. Binding potential (*BP*) of AMP 1018-K6 towards multilamellar vesicles of biological membranes.

Biological membranes	*BP*	
	Fluorescence	UV-Vis absorption
Hepatocytes	7.22	18.61
Salmonella spp.	26.87	18.78
Escherichia coli	10.71	42.35
Staphylococcus aureus	31.05	23.42
